# A Treatise on Corns, Bunions, and Diseases of the Nails

**Published:** 1845-07

**Authors:** 


### A Treatise on Corns, Bunions, the Diseases of Nails, and the General Management of the Feet. By *Lewis Durlacher*, Surgeon Chiropodist (by Special Appointment) to the Queen. Octavo, pp. 196. London, 1845. Simpkin, Marshall and Co.

Some medical men may affect to despise so humble a branch of the healing art as the treatment of Corns and Bunions. Not so Sir B. Brodie, who published some years ago two or three excellent lectures on the subject; nor our English Hippocrates, who did not hesitate to acknowledge that, in his opinion, *quantulamcunque in hoc scientiæ genere* (*morborum medela*) *accessionem, etsi nihil magnificentius quam Odontalgiæ aut Clavorum pedibus innascentium curationem edoceat, longe maximi faciendam esse, præ inani subtilium speculationum pompa ac levicularum rerum notitia, quæ fortasse medico ad abigendos morbos non magis ex usu futura est, quam architecto ad construendas ædes musicæ artis peritia.*"—Observat. Med. sect. 2.

Under such high sanction, let us proceed to notice the present volume.

Mr. Durlacher is a sensible man and a clever chiropodist. He has had the honour of *arranging*—that is the proper word—the toes and nails of three successive sovereigns of this kingdom. George the Fourth was much annoyed, we are informed, by the nail of the great toe on the left foot pressing into the flesh: Mr. D. waited upon his Majesty until six days previously to his death! Good King William's "toes were deformed and tender, so that he was continually complaining of them, but nothing could be found to afford relief." Our gracious Queen and Governor is (we trust) exempt from all pedal annoyances; but ladies are so invincibly fond of small shoes, that we should not be at all surprised to hear that the aid of her special Chiropodist was one day called into requisition.

Mr. Durlacher, like every other man who will tell the truth, admits that corns are invariably produced by the pressure of tight or ill-made shoes and boots, and that most persons might readily get rid of them if they would but consult their comfort at the expense of fashionable appearance. Wellington boots are, in our opinion, the worst of all; they confine the feet and legs, and are usually made more pointed at the toes than any other variety of *chaussure*. Since we have taken to the use of cloth-boots, *our* toes have been vastly more comfortable.

Mr. D. recommends that the feet should be washed every morning and evening; but he afterwards qualifies this advice by saying, that "adults in good health may bathe their feet every morning with cold water, wipe them thoroughly dry afterwards, and then rub Eau de Cologne freely over them with the palm of the hand. When dressing for dinner, the feet should be washed with soap and water in the same manner as the hands." The too-common custom of bathing the feet in warm or hot water is very hurtful: ladies especially do themselves much harm in this way.

The practice of our author seems to be judicious, and quite free from all quackery. The remedies, which he uses, are few and simple; being chiefly "the nitrate of silver, cold water, spirit lotions, and soap-plaster."

As a matter of course, the hard skin must be picked or cut off, before these applications can be of any use. He ridicules the charlatanerie of those advertising chiropodists who delude the public by asserting that they possess an infallible nostrum, capable of thoroughly eradicating corns!

Mr. Durlacher thus notices one or two of the less frequent forms of the complaint.

"A very troublesome corn is found under the great toe-nail. It is generally the result of accident, or from the nail having been allowed to grow to too great a length, so that it becomes compressed, or otherwise injured, against some hard substance, bruising the soft parts beneath, and thus producing a corn from extravasation. The patient is seldom aware of the period at which the injury was inflicted, as the corn is slow in its growth. When fully developed, a black or deep red spot is clearly visible through the nail, and is the seat of severe pain. As this corn increases in size it gradually loosens the nail, which is easily removed as far as the seat of the disease.

"Another form of corn is produced inside the inner fleshy flap of the great toe, extending in many cases under the edge of the nail. It is caused by the nail having been improperly cut, or by the first toe pressing against the flap, and pushing it up higher than ordinary, so that the inner cuticle becomes thickened in layers, as a protection against the sharp edge of the nail. The corn is formed under these layers, several of which must be removed before it can be brought into view, or extracted. Until it is fully developed, no pain is experienced." P. 13.

"In operating on corns growing under the great toe-nail, the first thing to be done is the removal of as much of the nail as is loose, which can be effected with very little pain, with an ordinary nail-knife, pair of scissors, or nail-nippers, by first slitting it up on each side with the nippers or scissors, and removing the portion of nail thus partially separated with the knife, so as to expose the corn, which is then to be extracted in the usual way.

"This form of corn, being produced by accidental causes, when properly extracted seldom or never returns.

"In those cases, where there is a corn situated in the inner flap of the great toe, it is necessary to remove several layers of the cuticle, previously moistening them, until the corn is exposed, when it should be extracted. If it extends beneath the nail, a part thereof must be cut away, until the corn is distinctly seen, and then it should be taken out." P. 15.

*Soft corns* are of the same structure as the hard ones. They "are always situated between the toes, and derive their name from being constantly in a state of moisture, occasioned by the perspiration or exhalation which collects between the toes, and is condensed within the cuticle on some prominent point where pressure has produced a corn.

"Soft corns are not deep-seated, and do not project much above the surface, on account of the structure of the parts, and the compression they are subject to.

"They are generally caused by the bone of one toe being pressed againt the opposite joint, or by the second phalanx or joint of the little toe being forced down upon the metatarsal bone of the third. All prominent parts on the inner side of the toes are liable to this formation."

Ladies are most subject to this variety of corns, in consequence of the absurdly-narrow shoes which they delight to wear. Their formation often commences in a little blister, caused by the pressure of one toe against another: when the serum is discharged, the new epidermis thickens into layers, and a corn is produced.

There is nothing peculiar in the treatment of soft corns. The thickened cuticle should be removed to as great a depth as it presents a whitish appearance; and, if a distinct corn has formed, it should be extracted. The Nitrate of Silver may then be slightly rubbed over its surface, and the toes kept as much apart as possible, so as to prevent the cause of its re-formation.

"I have met," says our author, "with a peculiar form of soft corn, where chalk has been secreted beneath it. This disease is of rare occurrence, and attacks those persons only who are subject to gout; it is attended with considerable pain and annoyance." P. 48.

The feet and toes are occasionally the seat of Neuralgic suffering. The following description is given by our author.

"There is another disease affecting these parts of the toes, which, although not absolutely a soft corn, should be noticed here, as it may be mistaken for that complaint by persons who are subject to it. It is a kind of neuralgia seated between the toes, but which fortunately is not very common. It constitutes a most troublesome and severe complaint, and one very difficult of removal.

"The patient complains of a severe pain between two of the toes, along the inside of one or the other, generally the second and third, he can seldom tell which; it extends up the leg, and is increased when the toes are pressed together, more particularly after walking. Notwithstanding the most careful examination of the part, no obvious cause can be discovered for the pain, and like all similar affections of the nerves, there is not any remedy to be depended upon, as it appears to defy all medical treatment." P. 49.

"Another form of neuralgic affection occasionally attacks the plantar nerve on the sole of the foot, between the third and fourth metatarsal bones, but nearest to the third, and close to the articulation with the phalanx. The spot where the pain is experienced can at all times be exactly covered by the finger. The pain, which cannot be produced by the mere pressure of the finger, becomes very severe whilst walking, or whenever the foot is put to the ground." P. 52.

A case is given in point: and, as the Chiropodist seems to have been a better doctor upon the occasion than the titled physician, we give it for the benefit of our author.

"I was requested by Sir James Clarke, Bart., to call on a lady who was suffering from this complaint. She was of delicate health, and appeared to be labouring under hysteria. She had so great a dread of the pain, that she dared not place her foot to the ground, and was consequently prevented from taking any other than carriage exercise. I placed a piece of adhesive plaster firmly round the foot, and then stated to Sir James Clarke that my further attendance was not necessary, as the disease was entirely constitutional." P. 53.

These neuralgic complaints of the toes are probably akin to what has been called the Writer's Cramp of the fingers.

Under the name of Neuro-vascular Corns, Mr. Durlacher describes "a peculiar and painful species of corn, having villi or nervous fibrillæ (!) clearly visible, running in zigzag whitish lines within the induration, and small corns appearing between them like white specks, corresponding in form to the cells or follicles they occupy." The expression "nervous fibrillæ" must surely be quite inappropriate here. The treatment of these corns will doubtless require greater tact and caution, in consequence of their extreme sensitiveness. When they occur in old people, they should not be irritated or much tampered with.

The term *Bunion* should be restricted to designate an enlargement or thickening of the integuments—the part being often studded with clusters of small superficial corns—over the first joint of the great or little toe, at the articulation with the metatarsal bone. It seems to be derived from the French word "oignon,"—which is still used among our neighbours for the complaint. When a Bunion becomes irritated by pressure, the subjacent bursa very frequently becomes enlarged, and serum is effused into its cavity. "If the disease still proceeds, the pain and swelling continue to increase, and suppuration takes place within the cavity of the bursa, which, on account of the depth of its situation and the abnormal thickening of the integuments, is very slow in bursting externally. Sometimes the ichorous fluid burrows into the adjoining cellular tissue, undermining the periosteum, and in some cases causing caries of the bones, with ulceration and exfoliation of the joint."

The treatment of Bunions is very nearly the same as that of Corns.

"The most beneficial and proper local remedies I generally use are cold-water dressings, spirit lotions, and the application of diachylon and soap plasters; in the more severe cases, linseed-meal poultices, made with decoction of poppies, the nitrate of silver, potassa fusa, and also nitric acid, but these latter remedies must be employed with the greatest caution." P. 99.

If the subjacent bursa remain full and painful, its contents should be discharged by a small puncture, and the edges should be every now and then touched with the lunar caustic. The part should afterwards be covered with a piece of soap-plaster, and an easy boot worn, so as to avoid all pressure.

*Diseases of the Nails.*—These tegumentary appendages sometimes become so hard that they cannot be cut with scissors or even with nippers. We must then saw the projecting piece off with a fine small saw, "beginning on the upper surface, and gradually cutting towards the sides, the instrument being held rather obliquely, and care being taken not to cut through the nail at once, for fear of wounding the tuft of enlarged papillæ, which is frequently found under it: the occasional dropping hot water into the cut will assist the operation, by softening the nail."

For the relief of that most troublesome, and often most painful, disease, known as "the nail growing into the flesh," our author recommends (when simpler means do not suffice) the following plan :—

"The preventive method which I adopt, when the nail is rather thick and the curved edge has not injured the integuments, is as follows. I scrape the top of the nail where the curve commences, and divide the inner part which lies in the fold of flesh, by carefully slitting the nail on the upper surface from the apex, to as near the root as possible, with a properly made instrument, taking care not to cut through it so as to cause hæmorrhage; the piece thus divided is not to be removed, so that the flesh, by its pressure upwards, forces it gradually to lap over the body of the nail in such a manner, that it cannot penetrate the soft parts. As the nail grows forward to the apex of the toe, it becomes of sufficient length to be cut across with the other part; then the slit must again be carried lower down, and the nail scraped below the part which had been previously operated on. If this operation be properly performed, it can be done without giving pain; and I have known it frequently succeed when every other means have failed, and even prevent the nail from penetrating, when cutting out the piece has been ineffectual.

"When the nail has penetrated into the flesh, and ulceration has commenced, these palliative measures prove of but little service, even when practised before the appearance of fungus, and relief can only be obtained by the excision of the diseased part of the nail. This has been effected in various ways; the operation in general use in this country is that practised by the late Sir Astley Cooper, and consists in passing one limb of a strong pair of scissors under the nail, slitting it up to the root, and then pulling out the piece when detached with forceps." P. 137.

Not much need be said about the nails of the fingers, as they seldom give rise to any trouble. The following hint may be useful.

"When a splinter of wood, or any other substance, has been forced under the nail so far that it cannot be laid hold of with the forceps or tweezers, and yet is visible, the readiest means for its extraction, and that which is attended with the least pain, is to cut down upon it, by carefully removing a narrow wedge-shaped piece of the nail until the end of the substance can be taken hold of with the forceps. Cold water dressing should then be applied for a few hours, and the part afterwards protected with a piece of black caoutchouc sticking plaster." P. 158.

*Warts* are thus distinguished from corns. "In their structure they differ altogether from that of corns, as they arise directly from the true skin, and appear to be composed of an elongated bundle of its papillæ, enclosed in sheaths of cuticle, whereas corns are a disorder of the epidermis alone."

Nothing novel is said about their treatment.

Such readers, as are curious in the history of those subjects of which his volume treats, Mr. Durlacher recommends to "consult the works of Hippocrates, Celsus, Scribonius Largus, Oribasius, Octavius Horatianus Aëtius, Actuarius, Nonnus, Avicenna, Haley Abbas, Phases, and Alsaharavius!"

On the whole, our author's work does credit to his judgment and candour. The coloured lithographed drawings are exceedingly good.

